# Chemokine analysis as a novel diagnostic modality in the early prediction of the outcome of non-union therapy: a matched pair analysis

**DOI:** 10.1186/s13018-018-0961-4

**Published:** 2018-10-10

**Authors:** Patrick Haubruck, Anja Solte, Raban Heller, Volker Daniel, Michael Tanner, Arash Moghaddam, Gerhard Schmidmaier, Christian Fischer

**Affiliations:** 1 HTRG—Heidelberg Trauma Research Group, Center for Orthopedics, Trauma Surgery and Spinal Cord Injury, Trauma and Reconstructive Surgery, Heidelberg University Hospital, Schlierbacher Landstrasse 200a, 69118 Heidelberg, Germany; 2 Raymond Purves Bone and Joint Research Laboratories, Kolling Institute of Medical Research, Institute of Bone and Joint Research, University of Sydney, St Leonards, New South Wales 2065 Australia; 3 Department of Transplantation Immunology, Institute of Immunology, University of Heidelberg, Im Neuenheimer Feld 305, 69120 Heidelberg, Germany; 4 ATORG—Aschaffenburg Trauma and Orthopedic Research Group, Center for Trauma Surgery, Orthopedics and Sports Medicine, Am Hasenkopf 1, 63739 Aschaffenburg, Germany

**Keywords:** Bone regeneration, Non-union, Chemokine, Cytokine, Diagnostics, Prediction

## Abstract

**Background:**

Despite the regenerative capability of skeletal tissue fracture, non-union is common. Treatment of non-unions remains challenging, and early determination of the outcome is impossible. Chemokines play an important role in promoting the formation of new bone and remodeling existing bone. Despite their importance regarding the regulation of bone biology, the potential of chemokines as biological markers reflecting osseous regeneration is unknown.

The purpose of this study was to determine (1) if serum chemokine expression levels correlate with the outcome of non-union surgery and (2) if chemokine expression analysis can be used to identify patients at risk for treatment failure.

**Methods:**

Non-union patients receiving surgical therapy in our institution between March 2012 and March 2014 were prospectively enrolled in a clinical observer study. Regular clinical and radiological follow-up was conducted for 12 months including collection of blood during the first 12 weeks. Based on the outcome, patients were declared as responders or non-responders to the therapy. To minimize biases, patients were matched (age, sex, body mass index (BMI)) and two groups of patients could be formed: responders (R, *n* = 10) and non-responders (NR, *n* = 10). Serum chemokine expression (CCL-2, CCL-3, CCL-4, CXCL-10, CCL-11, and interferon gamma (IFN-γ)) was analyzed using Luminex assays. Data was compared and correlated to the outcome.

**Results:**

CCL-3 expression in NR was significantly higher during the course of the study compared to R (*p* = 0.002), and the expression pattern of CCL-4 correlated with CCL-3 in both groups (NR: *p* < 0.001 and *r* = 0.63). IFN-γ expression in NR was continuously higher than in R (*p* < 0.001), and utilization of CCL-3 and IFN-γ serum expression levels 2 weeks after the treatment resulted in a predictive model that had an AUC of 0.92 (CI 0.74–1.00).

**Conclusion:**

Serum chemokine expression analysis over time is a valid and promising diagnostic tool. The chemokine expression pattern correlates with the outcome of the Masquelet therapy of lower limb non-unions. Utilization of the serum analysis of CCL-3 and IFN-γ 2 weeks after the treatment resulted in an early predictive value regarding the differentiation between patients that are likely to heal and those that are prone to high risk of treatment failure.

**Electronic supplementary material:**

The online version of this article (10.1186/s13018-018-0961-4) contains supplementary material, which is available to authorized users.

## Background

Bone is one of the few tissues that can heal without a fibrous scar, thereby osseous healing is considered as a form of tissue regeneration [1]. The osseous healing cascade is a complex physiological process involving multiple parameters both on a molecular and cellular level [1, 2] that need to act concertedly. Aberrations in this biological process can result in delayed healing or in the development of a non-union [1]. Despite the regenerative capability of skeletal tissue, fracture non-union is a common (up to 30% of fractures fail to heal) and persistent complication [3, 4]. Treatment of non-unions remains a challenge in orthopedics and trauma surgery [4] while multiple treatment modalities have been introduced lately. The Masquelet therapy was established as a safe and clinically effective treatment modality in the treatment of large non-unions [5]. Despite studies showing satisfying clinical results subsequent to the Masquelet therapy [6, 7], early determination of the outcome remains impossible. At present, the outcome is usually assessed as early as 6 months after surgery based on radiologic findings that require ionizing radiation (computed tomography and X-rays) [8]. In addition, no valid marker exists identifying patients that are prone to high risk of treatment failure. Early identification of those patients at risk would assist treating physicians in the postoperative management and provide a rationale for adjunct non-union treatment or timely revision surgery.

Chemokines are a family of signaling proteins secreted by cells that are specific to vertebrates [9]. They can be assigned to two major subfamilies: CXC (C–X–C motif) and CC (C–C motif) chemokine [10]. Members of these subfamilies play an important role in bone biology [9] and promote bone formation developmentally and in response to mechanical stimuli [11]. In particular, they modulate the formation of new bone and remodeling of existing bone by coordinating cellular homing, osteoblastogenesis, and osteoclastogenesis [10]. Due to their important role regarding the regulation of bone biology, recent research focus has shifted towards several chemokines and their mechanisms of action associated with bone remodeling [10]. Thus, exploration of chemokines as biological markers reflecting osseous regeneration seems natural.

In previous studies [11, 12], the serum cytokine analysis was established as a valid method investigating into biological processes occurring during bone regeneration subsequent to non-union therapy. Hence, this study was aimed to determine primarily if serum cytokine expression levels of distinct chemokines correlate with the outcome of non-union surgery. Secondly, the possibility to determine a prognostic model regarding the outcome of non-union therapy based on the expression levels of chemokines was investigated. Due to their importance in bone healing, CCL-2, CCL-3, CCL-4, CXCL-10, CCL-11, and interferon gamma (IFN-γ) [9, 10, 13, 14] were included and analyzed. The hypothesis of the study was that the expression patterns of distinct cytokines correlate with bone regeneration occurring during non-union therapy and can be used to identify patients at risk at an early stage.

## Methods

### Study design

To answer the research questions, a prospective clinical observer study was performed. The study was conducted at the Department of Orthopedics and Traumatology at the Heidelberg University Hospital (a level 1 trauma center). A total of 207 patients suffering from long-bone non-union and receiving surgery between March 2012 and March 2014 in our department were enrolled in the study. Due to the highly sensitive chemokine measurement, strict inclusion and exclusion criteria were applied and ultimately patients were matched in order to reduce confounders and influences onto the results of this study. Inclusion of patients started after approval of the local institutional ethics committee (S-636/2011). In addition, the study was conducted in accordance with the latest version of the Declaration of Helsinki.

### Inclusion and exclusion criteria

Patients suffering from failed bone healing after diaphyseal fractures of the tibia or femur that were between 18 and 80 years old and gave a written declaration of consent were included in the study. Initially, patients that were unable or unwilling to give a written consent, suffering from chronic inflammatory diseases or malignancies, needed to take immunosuppressive medication, or suffered from renal or hepatic failure were excluded from the study. In addition, patients that required additional surgical interventions or re-revisions were excluded during the course of the study.

### Rationale for group assignment

Patients that failed to show consolidation within 12 months after the second step of the Masquelet therapy were determined as non-responders, whereas patients that showed proper consolidation were determined as responders. Based on the outcome, patients were matched based on three established criteria (age, sex, and BMI) [11] and two groups were formed:

1. Responders to the therapy (group: R/*N* = 10)
2. Non-responders to the therapy (group NR/*N* = 10)

If more than one match was found for a patient, then the patient with the most similar type of non-union was chosen (Table 1).

**Table 1:** Patient characteristics

Patients	All	Responders	Non-responders	Significance
Sex				
Male	12	6	6	*p* = 1.000
Female	8	4	4	
Age	50.75 ± 11.49	50.8 ± 13.05	50.7 ± 10.40	*p* = 0.4277
BMI	30.08 ± 6.57	27.92 ± 6.45	32.235 ± 6.25	*p* = 0.3946
Smoking				
S	8	3	5	*p* = 0.2865
NS	10	5	5	
FS	2	2	0	
Diabetes				
Yes	3	1	2	*p* = 1.0000
No	17	9	8	
Localization				
Tibia	10	7	3	*p* = 0.1797
Femur	10	3	7	
Fixation				
Nail	8	3	5	*p* = 0.6481
Plate	12	7	5	
Previous surgeries	3.15 ± 2.03	2.5 ± 1.08	3.8 ± 2.57	*p* = 0.7715

*S* active smoker, *NS* nonsmoker, *FS* former smoker; age is presented in years

### Intervention

According to the “diamond concept” [15], there are several core factors necessary to achieve fracture consolidation and bone regeneration [15] (vascularity, growth factors, mechanical stability, osteogenic cells, and osteoconductive scaffolds). The Masquelet therapy, also called induced membrane technique, was specifically designed to treat challenging non-unions [5] by enhancing local bone biology and inducing osseous regeneration via two steps [5, 16]. In the initial surgical treatment (step I), the non-union tissue, surrounding avital bone and avital surrounding soft tissue, is debrided leaving a defect site. In the same surgery, this defect is filled with polymethyl methacrylate (PMMA) that is impregnated with antibiotics. The emerging foreign body reaction induces the vascularized Masquelet membrane [17]. Harvesting of tissue samples occurs during the first step that is subsequently microbiologically examined. The first step is repeated until asepsis is achieved and guaranteed by negative microbiological results, and afterwards, the spacer is left in situ for 6 weeks to enable a fully grown Masquelet membrane [18]. In a second step, the spacer is removed while leaving the membrane intact and the defect site is filled with a combination of autologous bone graft and additional growth factors (3.3 mg of bone morphogenetic protein 7) [5, 18–20]. De novo osteosynthesis is performed during the first or second step based on the anatomical localization and morphology of non-union. Thereby, the Masquelet therapy provides all factors necessary for bone healing according to the “diamond concept.”

### Postoperative care and determination of outcome

According to previously published protocols [6, 11, 12], clinical and radiologic examination was performed as part of a dedicated follow-up program. In addition, patient data was thoroughly assessed preoperatively and during each follow-up visit. Examination occurred prior and 2 days as well as 1 week subsequent to each step. In addition, examination was performed 2, 4, and 6 weeks, as well as 3, 6, and 12 months, after the second step. Blood samples were collected until 3 months after the second step of the treatment (Additional file 1: Figure S1). Patients included in the study were completing most of all follow-up examination. However, due to unavailability, occasionally, single isolated blood samples were not obtained. Outcome was evaluated 12 months after the final surgical treatment and based on radiologic signs of consolidation (bridging in three out of four cortices in conventional X-rays) and mechanical stability and full weight-bearing [21–23].

### Sample acquisition and measurement

Venous blood samples were taken (S Monovette 7.5 ml, Sarstedt AG, Germany) from all patients following a highly standardized previously published protocol [12]. Analysis of C-reactive protein (CRP) and leukocytes was conducted directly after the blood was drawn. The quantitative analysis was performed with Luminex Performance Human High Sensitivity Assays (Quantikine®, RD Systems, Minneapolis, MN, USA) strictly according to the manufacturer’s instructions. The lab technician performing the Luminex assays was blinded to both patient data and clinical outcome.

### Determination of sample size

Prior to commencement of the study, sample size determination was performed based on previously published data [24]. In particular, the sample size calculation for this study was performed in R [3.2.3] using the package “pwr.” Assuming an alpha level of .05 and a power of .80 as well as an equal number of subjects in the experimental and control groups, 9.41 patients per group were estimated to be required. Thus, a total of 10 patients per group were included.

### Patients demographics

**Fig. 1:**
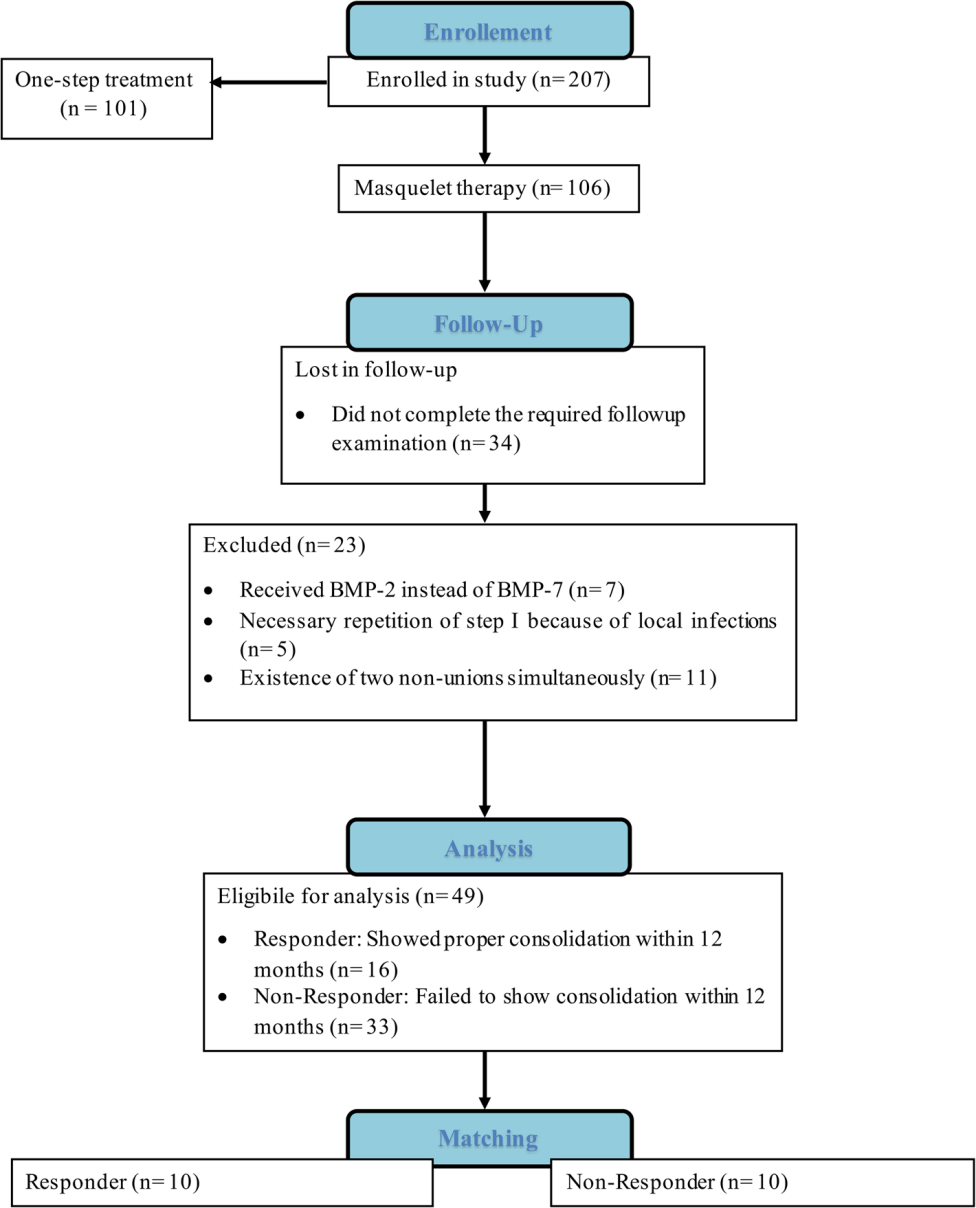
Flow diagram of the patient selection and exclusion process

Forty-nine patients were eligible for the current study (Fig. 1). According to our established matched-pair analysis, a total of 20 non-union patients were included into the current study (8 females and 12 males). Included patients were an average of 50.8 ± 11.5 years old. Statistically, patients of both groups had resembling characteristics regarding gender, age, BMI, smoking habits, diabetes, localization of the non-union, nail or plate fixation, and count of previous surgeries. Further details regarding patient characteristics in each group can be found in Table 1.

### Statistics

Explorative correlation analyses were conducted between all cytokine variables. Nonparametric test methods were assessed to investigate location shifts between groups (Mann–Whitney *U* test). Categorical variables were evaluated using the chi-square test. In order to assess if analysis of the expression pattern of the measured chemokines was able to predict the outcome of the therapy, multiple binary logistic regression models were utilized. Patients with incomplete data points were excluded from this analysis. Variables included were standardized. All initially available clinical variables (e.g., sex, age) as well as any serum parameters were included in the process to ensure valid assessment of the additional predictive power of the remaining potential covariates. Model selection was performed via AIC (Akaike information criterion) comparison. Predictive performance was assessed by estimation of the AUC (area under the curve) of the ROC (receiver operating characteristic) curve and the corresponding confidence interval. All *p* values quoted are to be interpreted in a descriptive way as they were not adjusted for multiple testing as this is an exploratory post hoc analysis. All statistical calculations were performed with R version 3.2.3 [25]. Figures were created by using the package “ggplot2” [26]. Serum levels are expressed as absolute mean concentrations ± SEM (standard error of mean), and statistical significance was determined as *p* < 0.05.

## Results

### Evaluation of inflammatory response

Independent inflammatory markers (CRP and leukocytes) revealed a physiological expression pattern without significant differences between groups. Both CRP and leukocyte count returned to normal 4 weeks after surgery (Table 2).

**Table 2:** Analysis of independent infectious parameters

Group	Measurement	Time point							
		Pre-op step I	2 days	1 week	Pre-op step II	2 days	1 week	2 weeks	4 weeks
Responders	L (mean ± SEM in 1000/μL)	7.434 ± 0.56	8.376 ± 0.83	7.331 ± 0.91	7.17 ± 0.71	8.156 ± 1.11	7.54 ± 0.89	7.285 ± 1.66	7.005 ± 1.01
	CRP (mean ± SEM in mg/L)	8.74 ± 2.85	100.4 ± 12.82	32.35 ± 9.96	14.32 ± 4.99	97.3 ± 14.89	47.29 ± 10.56	38.25 ± 12.70	8.05 ± 6.05
Non-responders	L (mean ± SEM in 1000/μL)	7.126 ± 0.52	7.391 ± 0.70	6.232 ± 0.47	6.559 ± 0.65	7.223 ± 0.70	6.952 ± 0.83	7.877 ± 1.50	5.53 ± 1.08
	CRP (mean ± SEM in mg/L)	9.06 ± 3.01	91.06 ± 12.44	35.33 ± 8.81	15.178 ± 9.06	105.275 ± 18.46	48.1 ± 8.72	15.033 ± 6.07	19.15 ± 13.95

### Evaluation of CCL-3 and CCL-4 serum expression

**Fig. 2:**
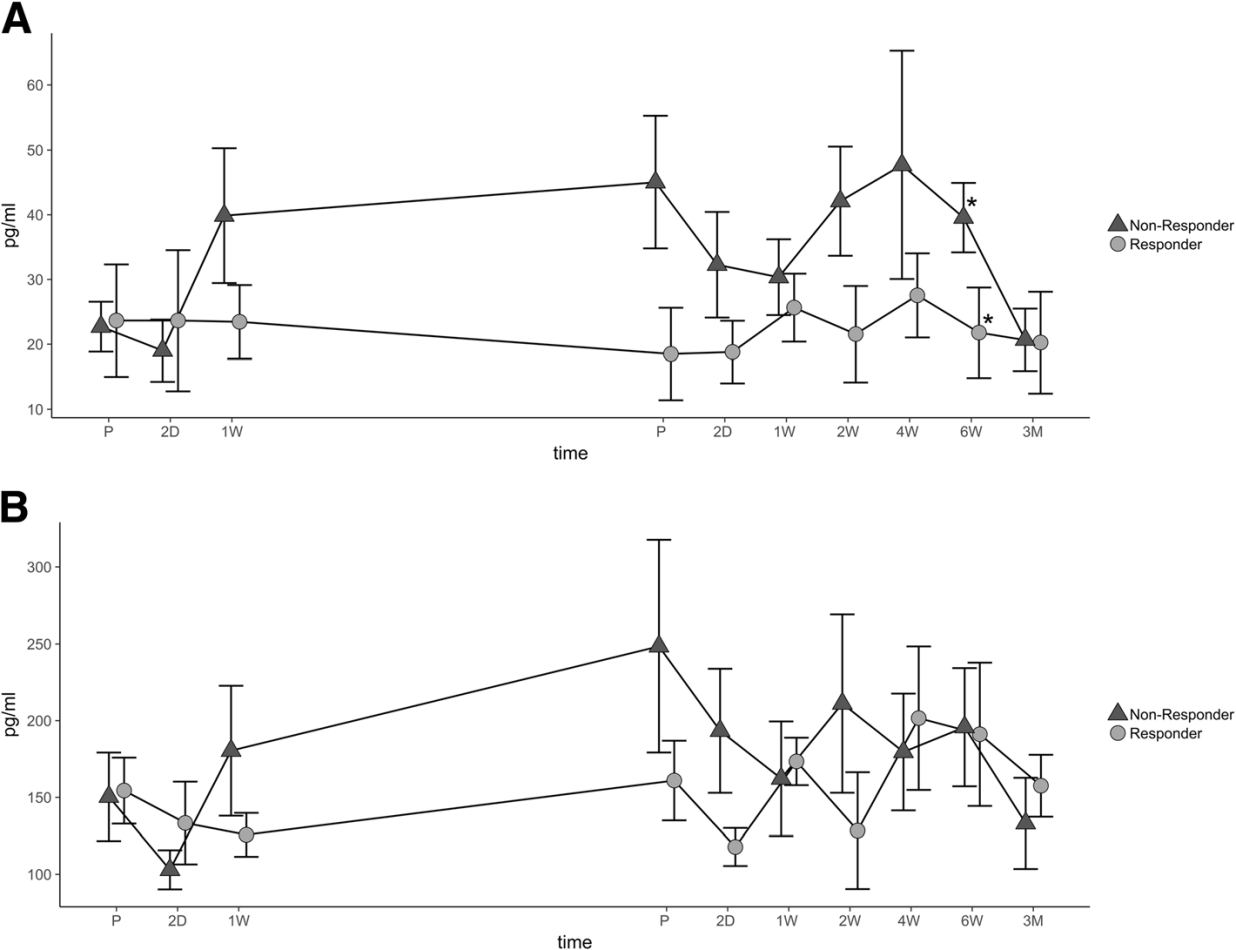
Analysis of CCL-3 and CCL-4. The average concentration and SEM (pg/ml) of CCL-3 (**a**) and CCL-4 (**b**) are shown during both steps of treatment and follow-up. Dark triangles display non-responders, and gray points indicate responders. Significant differences are indicated by a star (*p* < 0.05). Abbreviations: preoperatively (P), postoperative days (D), and weeks (W)

Following baseline expression, serum levels of CCL-3 in R slightly decreased until reaching a minimum prior to step II (18.51 ± 4.46 pg/ml); afterwards, expression in R was continuously lower than in NR reaching its peek 4 weeks after the treatment (27.54 ± 6.01 pg/ml). Starting 2 days after the initial surgery, expression of CCL-3 in NR was higher than in R throughout the study period. Analysis showed that expression of CCL-3 6 weeks after the treatment was significantly higher in NR compared to R (*p* = 0.036). Additionally, combined expression of CCL-3 in NR was significantly higher during the course of the study compared to R (*p* = 0.002 (Fig. 2a)). The expression pattern of CCL-4 was similar to the expression pattern of CCL-3 in both groups. Differences of CCL-4 between groups were at a nonsignificant extent (Fig. 2b). Expression pattern of CCL-3 and CCL-4 correlated significantly in NR to the therapy at 1 week after the first step (*p* = 0.001), prior to the second step (*p* = 0.047), and two days (*p* = 0.013) and one week after the second step (*p* = 0.040).

### Analysis of CCL-2 and CCL-11 serum expression

**Fig. 3:**
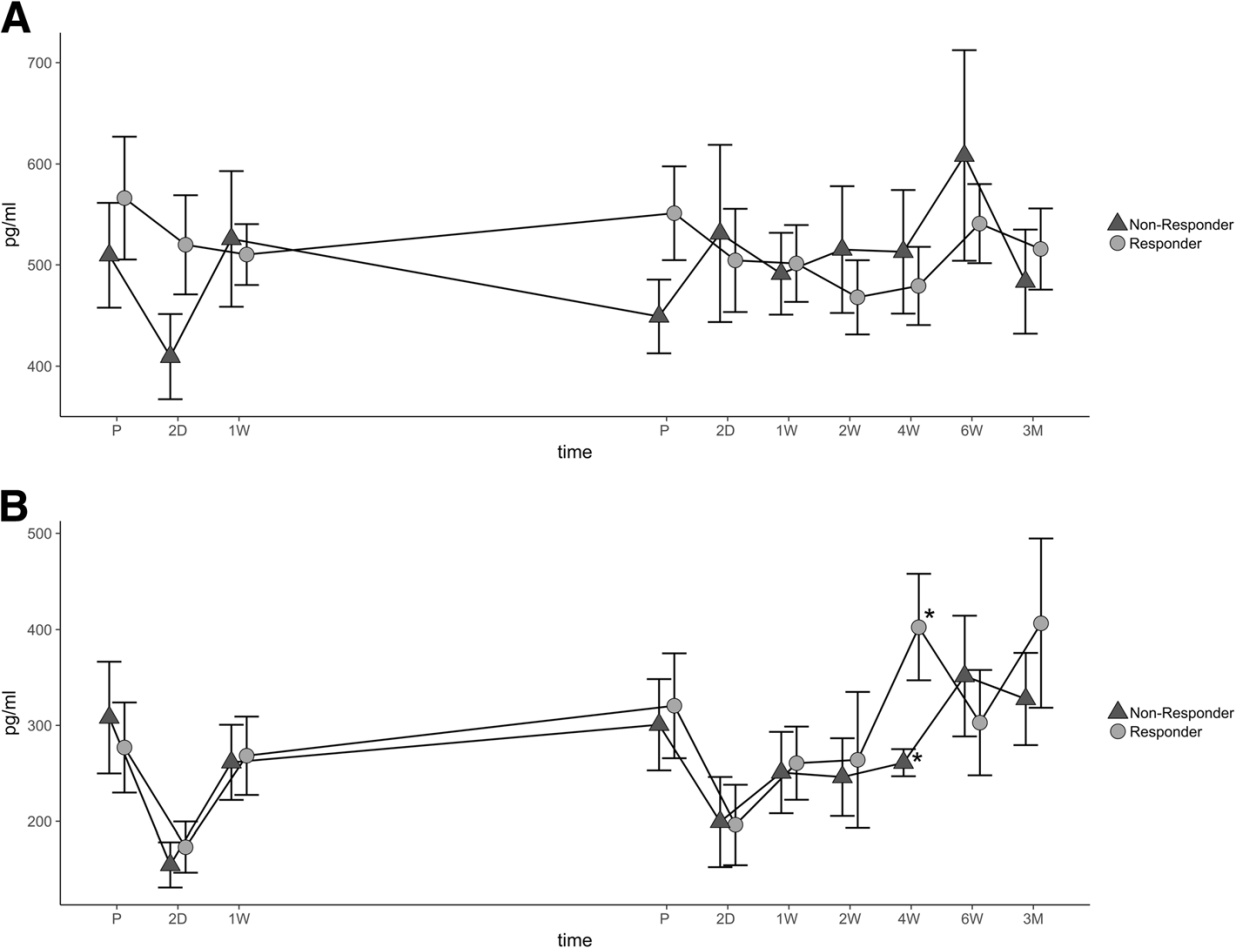
Analysis of CCL-2 and CCL-11. The average concentration and SEM (pg/ml) of CCL-2 (**a**) and CCL-11 (**b**) are shown during both steps of treatment and follow-up. Dark triangles display non-responders, and gray points indicate responders. Significant differences are indicated by a star (*p* < 0.05). Abbreviations: preoperatively (P), postoperative days (D), and weeks (W)

In R, the CCL-2 values were the highest prior to the surgical treatment (preoperatively, 566.21 ± 56.35 pg/ml) and the lowest two weeks after the second step (467.85 ± 32.90 pg/ml). In contrast, mean CCL-2 values in NR showed a minimum immediately after the first step (409.29 ± 39.82 pg/ml) and peak serum levels were reached 6 weeks following step II (608.23 ± 97.41 pg/ml). Statistical analysis revealed that differences between groups were at a nonsignificant extent during the course of the study. Interestingly, similar to CCL-3 and CCL-4, baseline expression of CCL-2 in R was higher compared to NR (Fig. 3a). Four weeks after step II, serum values of CCL-11 were significantly higher in responders to the therapy (R, 402.45 ± 51.24 pg/ml vs. NR, 261.06 ± 12.65 pg/ml; *p* = 0.047). Interestingly, peak values of CCL-11 in R were reached 3 months after the treatment (406.54 ± 83.07 pg/ml), whereas peak values in NR occurred 6 weeks after the treatment (351.51 ± 58.93 pg/ml) (Fig. 3b).

### Analysis of CXCL-10 and IFN-γ serum expression

**Fig. 4:**
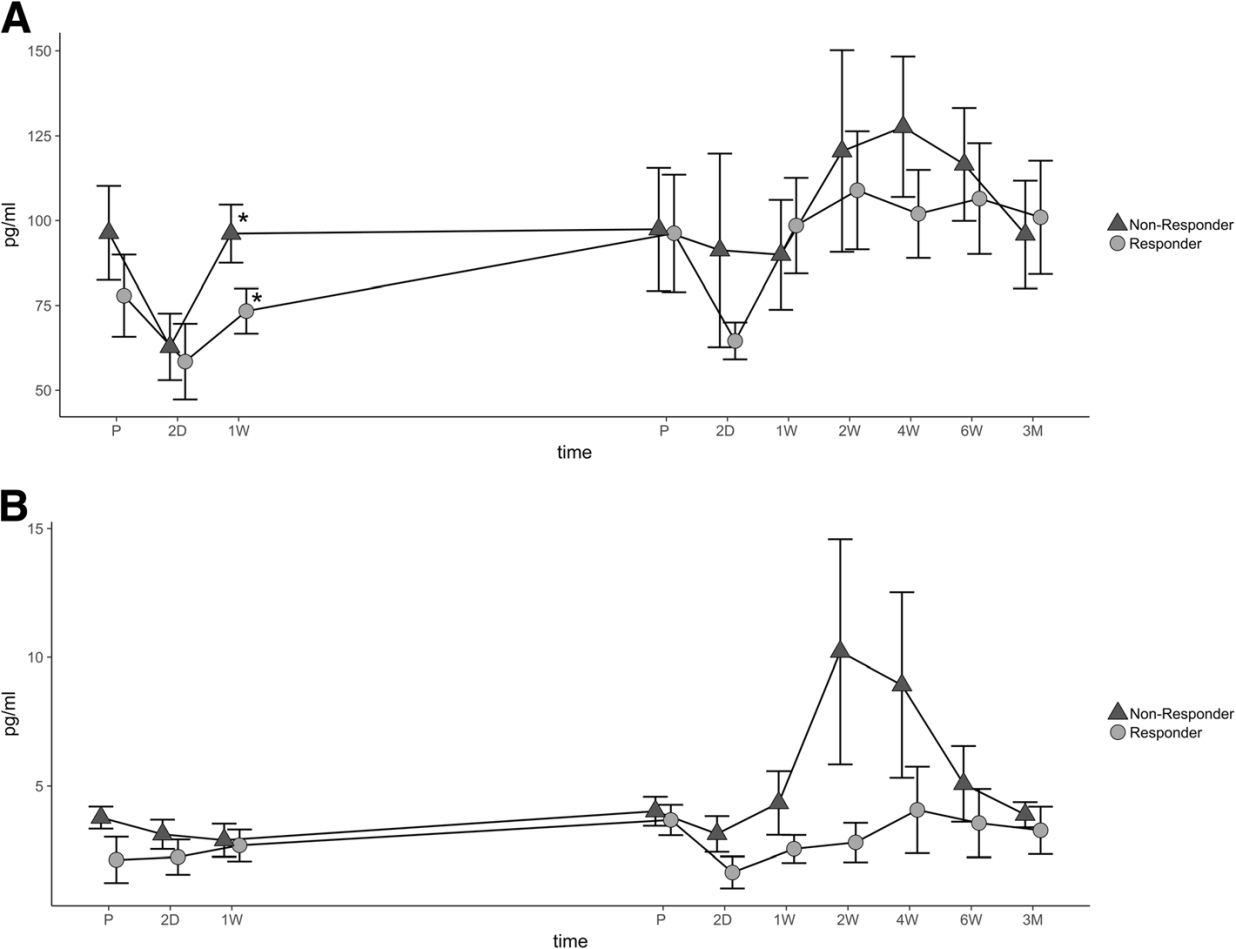
Analysis of CXCL-10 and IFN-γ. The average concentration and SEM (pg/ml) of CXCL-10 (**a**) and IFN-γ (**b**) are shown during both steps of treatment and follow-up. Dark triangles display non-responders, and gray points indicate responders. Significant differences are indicated by a star (*p* < 0.05). Abbreviations: preoperatively (P), postoperative days (D), and weeks (W)

CXCL-10 expression was slightly lower in R compared to NR and differences being significant 1 week after the initial treatment (*p* = 0.043). Expression was lowest in both groups immediately after step I (R, 58.45 ± 10.33 pg/ml vs. NR, 62.81 ± 9.31 pg/ml). Interestingly, expression in R showed a minimum immediately after each surgical treatment, whereas expression in NR only showed that minimum after the first step. Expression in NR peaked 4 weeks after step II (127.65 ± 18.49 pg/ml) and in R 2 weeks after the second step (108.94 ± 15.59 pg/ml) (Fig. 4a). Peak expression of IFN-γ in NR was higher (2 weeks after the treatment: 10.21 ± 3.91 pg/ml) compared to the peak reached in R (4 weeks after the treatment: 4.07 ± 1.56 pg/ml). Afterwards, values decreased until the end of the study. Combined expression of IFN-γ in NR was significantly higher than in R (*p* < 0.001) (Fig. 4b).

### Binary logistic modeling regarding the predictive power

**Fig. 5:**
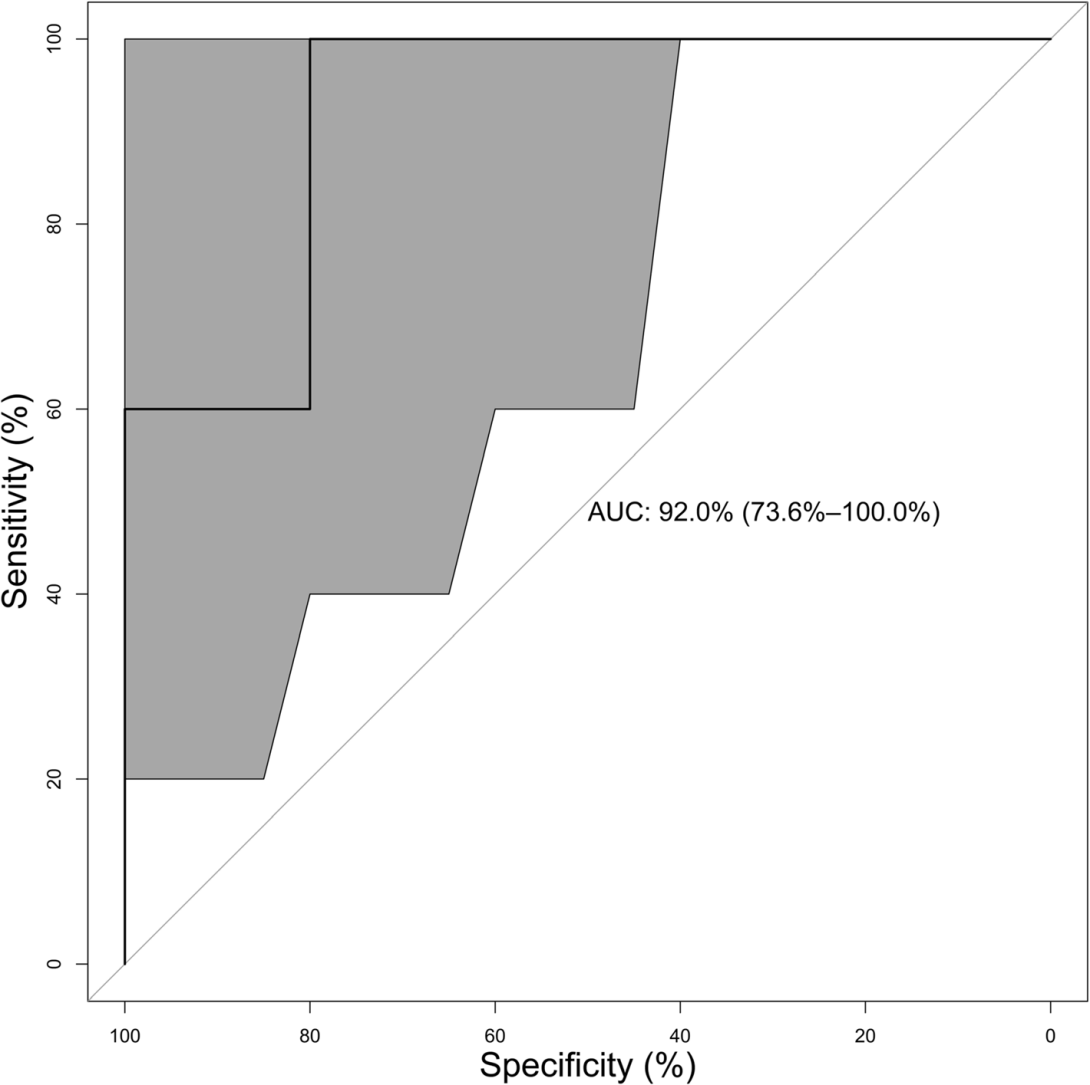
ROC of the predictive model. ROC of the binary regression model evaluating the predictive capabilities of the analysis of serum levels of CCL-3 and IFN-γ 2 weeks after the second step. 95% CI marked as gray area in both directions

Statistical analysis revealed that the best performing model was the one including only CCL-3 and IFN-γ serum expression levels 2 weeks after the second step of the Masquelet therapy. Regarding its predictive capabilities, the utilized model had an AUC of 0.92 (CI 0.74–1.00) and the resulting ROC is depicted in Fig. 5.

## Discussion

The findings of the current study provide important information regarding both research questions. Serum chemokine expression analysis over time of treatment is a valid and promising novel tool in the analysis of the expression pattern of distinct chemokines in context with non-union therapy. This data indicates that the chemokine expression pattern correlates with the outcome of the Masquelet therapy of lower limb non-unions. Ultimately, the analysis of CCL-3 and IFN-γ 2 weeks after the second step was able to identify patients that are at high risk for failure of the treatment.

The initial phase of fracture healing and bone regeneration is characterized by its inflammatory character [1]. CCL-2, also called monocyte chemotactic protein-1 (MCP-1), and its receptor CCR2 have been shown to induce the early inflammatory phase of tissue healing [27] and therefore play an important role during the early phase of fracture healing [27, 28]. Deletion of CCR2 only in the early phase of fracture healing has caused delayed fracture healing indicating the importance of increased CCL-2 expression for normal fracture healing [27]. In the current study, NR showed a minimum subsequent to the initial surgical treatment, whereas values in R only slightly decreased. The first step of the Masquelet therapy is intended to induce a vascularized membrane via a foreign body reaction [18, 29]. Interestingly, the foreign body reaction is initiated by an inflammatory response to the biomaterial similar to the response in early fracture healing [30]. Lower levels of CCL-2 after the first step in NR might correlate with an abnormal initial inflammatory response during the enfolding foreign body reaction that influences the outcome of the following bone regeneration.

Macrophage inflammatory protein 1 (MIP1) is a chemokine subfamily consisting of four members [31]. Relevant roles of CCL-3 expression, also called macrophage inflammatory protein 1 α (MIP1α), have been described for a variety of diseases [31]. However, no evidence exists regarding the role of CCL-3 during bone regeneration. In this study, beginning 1 week after the initial treatment, CCL-3 levels were constantly higher in NR. This elevated expression of CCL-3 might correlate with an increased osteoclastogenesis and active bone degradation resulting in an unfavorable microenvironment regarding the integration of the bone graft. This postulation is supported by findings from the literature. Expression of CCL-3 has been described in context with osteoclastogenesis and osteolysis [32]. Additionally, elevated expression of CCL-3 can be found in aseptic implant loosening and osteomyelitis [32] providing evidence of CCL-3 inducing differentiation of monocytes to bone-resorbing osteoclasts [32]. In particular, strong evidence exists regarding CCL-3 expression correlating with bone degeneration in patients with multiple myeloma providing proof regarding a link between CCL-3 activation and bone degradation [32–34]. Biological function of CCL-4, also called MIP1β, is closely connected to CCL-3 [35]. Studies have shown that CCL-3 and CCL-4 are predominant factors responsible for the enhancement of bone resorption in multiple myeloma [36] and play a causal role in the development of lytic bone lesions in vivo. In the current study, serum expression of CCL-4 correlated significantly with the expression pattern of CCL-3. Thus, expression of CCL-4 was higher in NR during most time of the study. This correlation supports previous findings by indicating that both CCL-3 and CCL-4 act in concert.

CCL-11, also called eotaxin-1, is a chemokine that is produced by a variety of cells including endothelial cells and chondrocytes [37]. In mice suffering from an inflammatory bone resorption, CCL-11 expression was higher compared to healthy controls [37], whereas in patients with chronic nonbacterial osteomyelitis, CCL-11 expression was lower compared to healthy controls [38]. Interestingly, the authors stated that expression of CCL-11 varied between different groups [38]. In bone regeneration subsequent to the Masquelet therapy, expression of CCL-11 was similar during the initial 10 weeks of the treatment. Hereafter, serum expression of CCL-11 was significantly higher in patients that responded to the therapy. Previous studies reported a chemokine-dependent amplification loop in bone metabolism [38, 39]. In particular, in aseptic conditions, CCL-11 was expressed by normal osteoblasts, while in inflammatory conditions, elevated CCL-11 levels stimulated migration of osteoclast precursors in addition to bone resorption [38]. Despite being initiated by an inflammatory response, bone regeneration in the current study occurred in aseptic conditions. Therefore, higher levels of CCL-11 in responders during bone integration might correlate with bone remodeling necessary to integrate the graft. However, the exact nature of this mechanism remains a speculation and is beyond the scope of this report.

The chemokine CXCL-10 is induced by IFN-γ resulting in its alternative name interferon-gamma-inducible protein 10 (IP-10) [40]. A relevant role for CXCL-10 was shown for chronic Th1 inflammatory diseases [40]. In particular, serum levels of CXCL-10 were elevated in patients suffering from a rheumatoid arthritis [40] and expression of CXCL-10 caused the recruitment of inflammatory cells and is involved in bone erosion in inflamed joints [41]. Interestingly, a recent study has shown increased CXCL-10 levels in the acute graft-versus-host disease (aGvHD) subsequent to bone marrow transplantation [42]. Thereby, measurement of CXCL-10 was postulated as possible biomarker in context with aGvHD [42]. In the current study, patients received autologous bone grafts and usually no graft-versus-host response is expected. However, a syndrome similar to GvHD has been reported to occur spontaneously in 8% of patients receiving autologous bone [43]. Interestingly, expression of CXCL-10 was elevated in non-responders in the initial 10 weeks of the treatment and peak levels were reached subsequent to the implantation of autologous bone graft. This elevated CXCL-10 might correlate with an abnormal response of the body to the transplanted autologous bone graft resulting in failure of the treatment. This altered host response regarding autologous bone might pose as an interesting approach in understanding factors influencing the outcome of bone regeneration and warrants further investigation.

IFN-γ is the only type II interferon and was originally associated with the host defense regarding a viral infection [44]. However, substantial evidence exists regarding the influence of IFN-γ onto bone healing. In particular, IFN-γ decreased the bone healing capability of mesenchymal stem cells (MSC) and MSC treated with IFN-γ underwent apoptosis [44]. Thereby, an anti-osteogenic function was associated with IFN- [44]. Another study showed that IFN-γ and tumor necrosis factor alpha synergistically induced MSC deficiency resulting in osteoporosis [44]. Autologous bone graft induces bone healing due to its osteoinductivity based on high concentrations of MSC [15]. In this study, initial expression of IFN-γ was similar during the first step of the treatment and only after the transplantation of autologous bone values were higher in non-responders to the therapy. These high levels of IFN-γ might inhibit the function of MSCs in the implanted graft causing an impaired osseous induction ultimately resulting in failure of the treatment.

Up to date, no valid biomarker exists capable of identifying patients that are at risk for an unsuccessful non-union treatment. Evaluation of the outcome relies on radiological diagnostics that require radiation exposure [8], and earliest determination is achievable 6–12 months postoperatively. Recalcitrant non-unions are associated with a low quality of life, long period of recovery, and strenuous surgical treatments that eventually fail. As traditional X-ray or CT scans are not able to determine the outcome at an early stage, the resulting uncertainty represents a considerable psychological burden for concerned patients. Thus, an early prediction of the outcome might contribute towards both an improved patient satisfaction and stratification of the postoperative management regarding the individual risk. In addition, early identification might provide rationale for additional postoperative non-union therapies, such as low-intensity pulsed ultrasound [45]. Results from this study introduced the measurement of CCL-3 and IFN-γ 2 weeks subsequent to the second step of the Masquelet therapy as promising novel diagnostic modality. In particular, evaluation based on these biological markers had a high sensitivity and good specificity in detecting patients at risk for poor outcome. Due to the commercial availability of the used Luminex assays, the low direct costs, and the short time necessary to perform the analysis, this method is easy to both implement and perform in all centers having a clinical laboratory. The promising results of the current study are intended to encourage surgeons to evaluate this diagnostic modality in their own setting. Ultimately, the findings of this study might contribute towards an improved patient safety in context with non-union therapy.

Despite relevant findings, our study has limitations. Non-unions are a severe and clinically relevant complication, whereas absolute numbers remain relatively small. In addition, serum cytokine and chemokine analysis are highly sensitive and can be influenced by various factors; therefore, a close matching of patients next to our strict inclusion and exclusion criteria was used to reduce the differences between groups. This explains the small patient collective of this study. In the context of current literature and recent studies [12, 46], the patient collective size is still sufficient to provide reliable results. Results of this study may be influenced by a systemic inflammation; therefore, the CRP and leukocyte serum patterns were assessed in the initial 4 weeks subsequent to the procedure. The data showed comparable CRP values during the course of the study in both groups; in addition, leukocytes remained in a physiological range during the whole time, thereby indicating that no systemic inflammation was present. The current study provided first evidence regarding the predictive capabilities of chemokine expression analysis in context with non-union therapy. However, further studies are needed that involve a larger non-matched patient collective in order to assess the influence of individual patient characteristics and establish a threshold for the introduced CCL-3 and IFN-γ test.

## Conclusions

The results of the current study introduce the serum analysis of the expression pattern of distinct chemokines as a novel diagnostic modality in context with bone regeneration occurring during non-union therapy. As bone regeneration occurs in the Masquelet therapy and the treatment is both highly standardized and regularly monitored, this treatment has become valuable in studying biological processes occurring during bone regeneration. The expression pattern of chemokines (CCL-2, CCL-3, CCL-4, CCL-11, CXCL-10, and IFN-γ) correlates with the outcome of the Masquelet therapy of lower limb non-unions. Furthermore, the analysis of CCL-3 and IFN-γ serum levels 2 weeks after step II of the therapy predicts the likelihood of successful induction of bone regeneration during the Masquelet therapy and provides an additional predictive value regarding the early identification of patients that are at high risk for failure of the treatment. Based on these results, the current study introduces an early predictive value regarding the differentiation between patients that are likely to heal and those that are at high risk for a poor outcome.

## Additional file

Additional file 1: **Figure S1.** Timeline of sample acquisition according to our standardized cytokine protocol. Clinical and radiological follow-up including acquisition of blood samples in blue. Additional sole clinical and radiological examinations in red. (PNG 5466 kb)

## Abbreviations

AIC: Akaike information criterion
AUC: Area under the curve
BMI: Body mass index
CCL: CC (C–C motif) chemokine
CI: Confidence interval
CRP: C-reactive protein
CXCL: CXC (C–X–C motif) chemokine
INF-y: Interferon-gamma
L: Leucocytes
NR: Non-responders
PMMA: Polymethyl methacrylate
R: Responders
ROC: Receiver operating characteristic
SEM: Standard error of mean
